# MicroRNAs reshape the immunity of insects in response to bacterial infection

**DOI:** 10.3389/fimmu.2023.1176966

**Published:** 2023-04-21

**Authors:** Muhammad Nadeem Abbas, Saima Kausar, Bibi Asma, Wenhao Ran, Jingui Li, Zini Lin, Tiejun Li, Hongjuan Cui

**Affiliations:** ^1^ State Key Laboratory of Resource Insects, Southwest University, Chongqing, China; ^2^ Cancer Center, Medical Research Institute, Southwest University, Chongqing, China; ^3^ Gastrointestinal Vascular Surgery, The Chongqing Ninth People’s Hospital, Chongqing, China; ^4^ Jinfeng Laboratory, Chongqing, China

**Keywords:** insects, host-pathogen interaction, microRNAs, innate immunity, immune pathway

## Abstract

The interaction between bacteria and insects can significantly impact a wide range of different areas because bacteria and insects are widely distributed around the globe. The bacterial-insect interactions have the potential to directly affect human health since insects are vectors for disease transmission, and their interactions can also have economic consequences. In addition, they have been linked to high mortality rates in economically important insects, resulting in substantial economic losses. MicroRNAs (miRNAs) are types of non-coding RNAs involved in regulating gene expression post-transcriptionally. The length of miRNAs ranges from 19 to 22 nucleotides. MiRNAs, in addition to their ability to exhibit dynamic expression patterns, have a diverse range of targets. This enables them to govern various physiological activities in insects, like innate immune responses. Increasing evidence suggests that miRNAs have a crucial biological role in bacterial infection by influencing immune responses and other mechanisms for resistance. This review focuses on some of the most recent and exciting discoveries made in recent years, including the correlation between the dysregulation of miRNA expression in the context of bacterial infection and the progression of the infection. Furthermore, it describes how they profoundly impact the immune responses of the host by targeting the Toll, IMD, and JNK signaling pathways. It also emphasizes the biological function of miRNAs in regulating immune responses in insects. Finally, it also discusses current knowledge gaps about the function of miRNAs in insect immunity, in addition to areas that require more research in the future.

## 1 Introduction

The innate immunity of insects is activated when pathogens that cause infections invade their bodies, which serves as a host defense system against microbial infections. The precise regulation of innate immune responses is crucial for maintaining immune homeostasis during microbial infection. It is also pivotal to prevent excessive immune responses that might result in tissue damage (1–4). The immune pathways of insects like Toll and Immune Deficiency (IMD) are evolutionarily conserved and play a critical biological role in the defense against microbial invaders. In insects, these pathways initiate the production of a wide range of antimicrobial peptides when fungal and bacterial infections occur, which are implicated as a mechanism of antiviral immunity (5–8). The Toll pathway controls the expression of the antifungal peptide, whereas the IMD pathway modulates the expression of the peptide that defends against Gram-negative bacteria (9–12). The expression of the genes that encode these peptides can, therefore, be used as a measure of the levels at which Toll and IMD signaling is activated. Although the Toll and IMD pathways are largely independent of one another, some cross-regulation occurs between them. In terms of the AMPs that are expressed *via* both pathways, Defensin has been demonstrated to be effective against Gram-positive bacteria. At the same time, AttacinA and Cecropin have been shown to be effective against Gram-negative bacteria (11, 13).

MicroRNAs (miRNAs) are a class of endogenously produced small (~22 nucleotides) non-coding RNAs that have been found to be evolutionarily conserved in nature and were first discovered two decades ago (14, 15). There is a lot of evidence that RNA polymerase II transcribes miRNAs, which are then processed by Drosha and Dicer, two nucleases. The RNA-induced silencing complex (RISC) incorporates and preferentially stabilizes functionally mature miRNA after it is exported from the nucleus into the cytoplasm. Generally, the RISC converts the miRNA into a complementary region of 6–8 nucleotides in the 3′-Untranslated Region of its target mRNA known as the ‘seed sequence’, which mediates its function. A miRNA that has partial or incomplete complementarity to a target mRNA may cause translation suppression. In contrast, a miRNA that has full or perfect complementarity to its target mRNA may promote post-transcriptional degradation levels. There is evidence that some miRNAs can bind to the 5′-UTR (Untranslated Region) and the coding sequences of the mRNA at which they are targeting, and many miRNAs can induce gene expression at the target locus (15–17). One mRNA is likely to be regulated by multiple miRNAs, and a miRNA can control the expression patterns of a diverse set of target mRNAs. As a result, it has become obvious that miRNAs play critical biological roles in a wide range of physiological activities, including cell immune responses, proliferation, differentiation, metabolism, and autophagy.

Since then, the biological role of miRNAs in various physiological processes, including their involvement in insect immunity against bacterial infection, has been established (15, 17). The importance of miRNAs in the regulation of innate immunity has recently received the attention of researchers all around the world. Therefore, in this review, to understand the importance of miRNAs in insect immunity against bacterial infection, we first summarize miRNA production and functional mechanisms. Also, we describe the deregulation of miRNAs that has been discovered during various bacterial infections. Following that, we discuss the host signaling pathways utilized by bacterial effectors that cause miRNA expression to be dysregulated in mechanisms of modulation by bacterial effectors, and we highlight the gaps in our understanding of miRNAs’ role in immunity to bacterial infections, as well as potential research areas that need to be explored in the future.

## 2 An overview of miRNAs in insects

The first miRNAs were discovered in 1993, and it has recently emerged as one of the more intriguing topics of research, with the number of studies published increasing exponentially over the last decade (18). In the same way that miRNAs have been described from different types of eukaryotic species, miRNAs have also been reported from various species of insects, especially those whose full genome sequence is known. As sequencing platforms improve, ongoing research has been able to identify new miRNAs and revise those that are already known. A number of studies on various insect species have demonstrated that miRNAs are implicated in a variety of physiological functions, including reproduction (19), metamorphosis (20), development (21, 22), sexual dimorphism (23), metabolism and longevity (24), cast determination (25), memory formation (26), behavior (27), insecticide resistance (28), endosymbiosis (29), and host–pathogen interactions and immunity (30), among others.

## 3 A brief description of the biogenesis and functional mechanism of miRNAs

**Figure 1:**
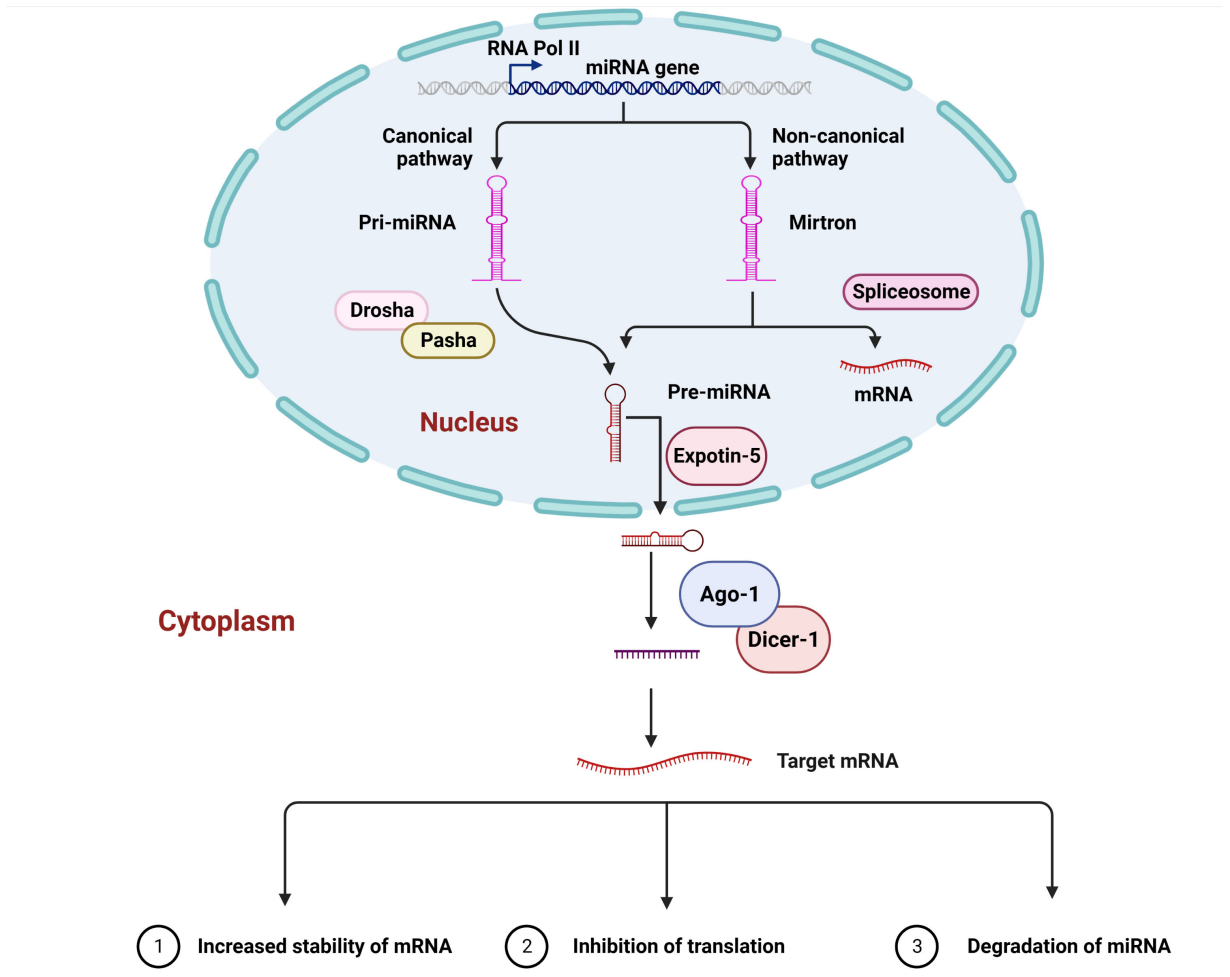
A canonical mechanism of microRNA biogenesis and their interaction. In living organisms, primary miRNA, like other cellular transcripts, contains a 5’ cap and a polyA tail. By cleaving the stem-loop at the base of the primary miRNA, the Drosha enzyme generates the precursor miRNA. Exportin-5 subsequently transports the precursor miRNA into the cellular cytoplasm. The Dicer-1 enzyme removes the hairpin head, resulting in the development of a miRNA duplex and, in turn, the production of the RNA-induced silencing complex, in which Ago1 is one of the crucial components. The miRNA passenger strand is generally degraded, whereas the guide strand is responsible for guiding the RNA-induced silencing complex towards the target mRNA, which is bound to either the open reading frame, the 5’ Untranslated Region, or the 3’ Untranslated Region. This interaction causes mRNA degradation, translation inhibition, and, in some cases, increased mRNA stability.

The general molecular mechanism underlying miRNA production in a cell is the same as the molecular mechanism underlying the production of coding-genes in that the miRNAs are transcribed through the enzyme RNA polymerase II in their primary form, which is polyadenylated and capped. The primary miRNA differs in length and comprises one or more stem-loop structures, which can be used as substrates by Drosha, a nuclear ribonuclease protein that, with the help of the Pasha protein, cleaves the stem-loop at the stem base. This results in releasing a hairpin structure containing 70 nucleotides and is recognized as the precursor miRNA (14). Following the precursor, miRNA is processed and transported from the nucleus into the cytoplasm *via* the nuclear membrane with the help of the Exportin-5 protein. Another ribonuclease enzyme (Dicer-1) is involved in removing the hairpin head in the cytoplasm. In contrast to vertebrates, insects have two proteins of Dicer: Dicer-1, which is implicated in miRNA biosynthesis, and Dicer-2t, which is involved in short interfering RNA generation (16). When the hairpin is removed, a duplex of RNA is formed, activating the formation of the RISC (RNA-induced silencing complex), a complex in which Argonaute 1 controls a critical function. The loaded RNA duplex is formed by two strands (passenger and guide strands). During the loading process, the passenger strand is degraded, and the guide strands are used to direct the RISC to target sequences based on the complementarity of the two sequences. The guide strand is referred to as miR-X-5p or miR-X-3p depending on whether it comes from the 3’ or the 5’ arm of the stem-loop, with X representing a designated miRNA number (**Figure 1**).

A key biological role of miRNAs has been to post-transcriptionally control the expression of genes *via* interacting with their target mRNAs so that they can fine-tune the level of protein in the cell (31). On the other hand, it has been demonstrated that miRNAs, which are localized in the cell nucleus, are capable of interacting with complementary sequences within the genome, resulting in either induced or silenced transcription depending on the context (17). The complementarity between miRNA nucleotide sequences and those of the target sequences differs; thus, it is expected that there may be mismatches between miRNA and target sequences. However, it is crucial for miRNA function that the seed region, which consists of 2–8 nucleotides from the 5’ end, has complementarity, while the lack of complete complementarity in the seed region of miRNA may compensate for the absence of complementarity in the rest of the sequence of the miRNA sequence (32, 33). It is believed that most of the effective binding sites for the miRNA can be found in the 3’ Untranslated Region (UTR). It has also been shown that miRNA binding can occur at the 5’-UTR or the ORF (31, 34, 35). For example, the results of a recent study that was based on the sequencing of hybrids technique ligation and cross-linking indicate that 42.6% of human DNA targets are located in the coding-region, 23.4% are located in the 3’-UTR, and 3.8% are located in the 5’-UTR (36). There is a similar level of biological targets found in the *Drosophila melanogaster* coding-regions to that found in the 3’-UTRs, which are very similar to those shown in the coding regions of the fly. A significant number of biological targets have also been discovered in the 5’-UTRs (35). However, it has been shown experimentally that target sites located in the 3’-UTR of genes are more likely to influence gene regulation in *D. melanogaster* than those located in the 5’-UTR or ORF of genes. It is also possible that the same or different miRNAs may bind to different binding sites in different parts of an mRNA, revealing multiple binding sites (37). It is interesting to note that the Ago1 cross-linking immunoprecipitation sequencing study was carried out on *Ae. aegypti* and published by Zhang et al. (38) reported that a number of potential target sites were identified in the coding-sequence of the Ago1. This implies that ORF-based targets may be common in other animals as well. There are two possible outcomes that result from miRNA–target interaction: degradation of mRNA or inhibition of translation (39–41). A growing body of research indicates that the interaction with the target can result in an increase in the abundance of the target as a result of the interaction. Despite the fact that the mechanism in the majority of cases is unknown, there are several possible explanations, including an increase in the stability of mRNA, or an increase in transcription, or an increase in translation (42–45). According to one possible explanation of why there are more examples of repression in the literature, it is possible that scientists have primarily studied the interactions between miRNA and targets with a bias towards repression when studying interactions between miRNA and targets.

## 4 Role of miRNAs in the regulation of insect-pathogen interactions

Because insects are a widely distributed group of animals that share a common environment with pathogens, they are more likely to become infected by these pathogens, which can have an enormous physiological effect on the living organism both at the organismal and cellular levels. In turn, the organismal body allocates extensive resources to inhibiting the replication of the pathogen and preventing it from spreading throughout the body by employing various immune defense mechanisms. Insects defense requires rapid modifications in the transcription of genes and alterations of proteins, which participate in the activation of immune pathways. It is worth noting that miRNAs, which are responsible for regulating the expression of genes, have also been linked to the responses of insects to infection. There is a growing body of evidence that miRNA expression profiles are altered following the invasion of a variety of pathogens, including bacteria, viruses, fungi, and protozoans when compared to uninfected insect expression profiles. There seems to be a wide range of alterations in immune responses, some of which may be triggered by microbial pathogens to assist their replication *via* compromising deference responses to the immune system or providing resources needed for their replication.

## 5 Bacterial infection changes the miRNAs of insects

**Figure 2:**
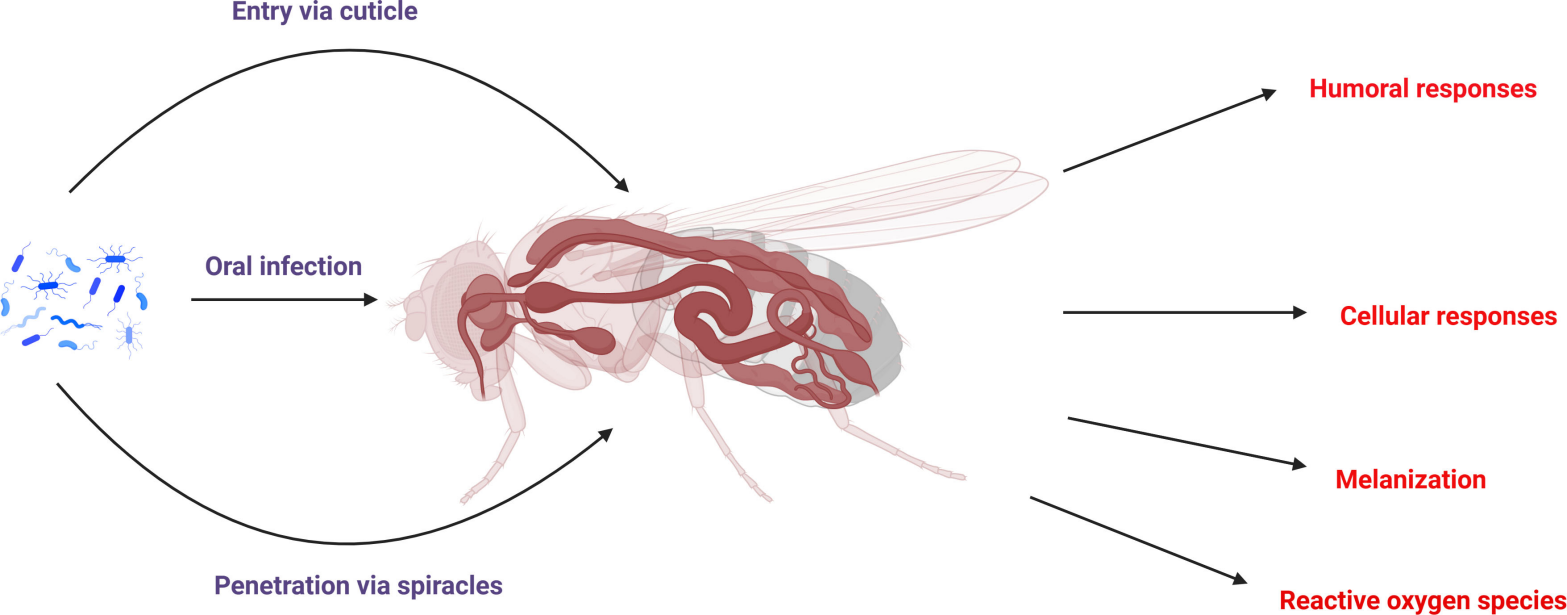
Schematic representation of the bacterial infection route in insects and also their major immune responses to bacterial infection.

Due to the advancement in the field of molecular techniques in the last few years, researchers have had the ability to study insect-pathogen interactions at much deeper levels than they were able to before, thanks to the discovery of these techniques, which allow them to study at a much more detailed level than previously possible. According to a growing body of evidence, the interaction between the host and bacterial pathogens (**Table 1**; **Figure 2**) is one of the most important research areas in the field of insect infection because bacterial pathogens interact with the host in a complex way. Aside from mRNAs, recent evidence suggests that the expression patterns of miRNAs have been altered by bacterial pathogens which invade insects, resulting in insect miRNAs exerting immense pressure on the bacterial pathogens. The following are some examples of bacteria that are known to be pathogens to insects and may have an influence on the expression profiles of insect miRNAs.

**Table 1:** miRNAs that play a biological role in the immune responses of insects to bacterial pathogens.

Insect	Pathogen	Tissue/cells	miRNA	Expression	Target gene or signaling pathway	Effects and remarks	Reference
*Drosophila*	*M. luteus*	Whole body	miR-959	Down↓	Tube	Inhibit Tube, thereby negatively regulating Toll signaling and AMPs production	(9)
			miR-960	Down ↓	Tube	Repress Tube, thereby negatively regulating Toll signaling and AMPs production	(9)
			miR-961	Down ↓	Dorsal	Suppress Dorsal, thereby negatively regulating Toll signaling and AMPs production	(9)
			miR-962	Down ↓	Dorsal	Inhibit Dorsal, thereby negatively regulating Toll signaling and AMPs production	(9)
	*M. luteus*		miR-375-3p	Up ↑	*Lsp2*	Regulate *Lsp2* gene expression, which is involved in the metabolic process, and it seems that this miRNA adjusts metabolism during the infection period	(46)
*Drosophila*	*M. luteus*	Adult flies	miR-958		Toll and Dif	Modulate Toll pathway *via* regulating Toll and Dif, thereby negatively controlling AMPs production	(47)
			miR-1017, miR-375, miR-4	?	Toll	In silico prediction showed that these miRNAs negatively regulate Toll activity. Experimental evidence is required for further confirmation	(47)
			miR-964, miR-137, miR-927	?	Pellino	In silico prediction showed that these miRNAs negatively regulate Pellino activity to inhibit the over-activation of the Toll pathway	(47)
			miR-1000, miR-981, miR-316	?	MyD88	In silico prediction showed that these miRNAs negatively regulate MyD88 activity to inhibit the over-activation of the Toll pathway	(47)
	*E. faecalis*		miR-317-3p	Down ↓	Dif-Rc	Negatively regulate the Toll signaling pathway and thereby control AMPs production	(48)
	*M. luteus*	Adult flies	miR-310, miR-311, miR-312 and miR-313	Up ↑	Drosomycin	These miR-310 family members can target and negatively modulate *Drosomycin* expression, an AMP produced by the Toll pathway	(49)
	E. coli	Adult flies	miR-9a and miR-981		Diptericin	These miRNAs possess different binding sites within the 3′UTR of *Diptericin*, the main effector gene induced by the IMD pathway. These miRNAs can also suppress AMPs by IMD pathway negative regulation	(50)
	*E*. *coli*	Adult flies	*miR-34*	Up ↑ with age	Dlg1, and Eip75B	The target genes are components of the ecdysone signaling pathway and a negative modulator of the IMD signaling pathway, thereby miR-34 controls AMPs	(51)
	*E*. *coli*		miR-277	Up ↑	*imd* and *Tab2-Ra/b*	dMyc inhibits *Drosophila* IMD immune response *via* directly activating *miR-277* transcription, which further inhibits the expression of IMD and *Tab2-Ra/b*	(52)
*P. xylostella*	*B. thuringiensis*		miR-1, miR-10, miR-184, miR-275, Let-7	Up ↑	?	?	(53)
			miR-2b-3p	Up ↑	Trypsin	Suppress the activity of trypsin	(53)
*Ae. aegypti*	*W. pipientis*		aae-miR-2940	Up ↑	metalloprotease gene	Regulate metalloprotease gene expression	(54, 55)
*G. mellonella*	*S. entomophila*	Midgut, and eggs	api-miR-263a	Up ↑		Involve in immune priming	(56)
*G. mellonella*	*L. monocytogenes*	Larval body	miR-998 and miR-133	Down ↓	optineurin	Likely negatively governs optineurin activity, however, further experimental evidence is required	(57)
			bmo-miR-3000	Up ↑	chitotriosidase-1 and cytochrome P450 6B4	Likely regulate the activity of chitotriosidase-1 and lysozyme, however, experimental evidence is required to confirm this relationship	(57)
			miR-954-5p	Up ↑	cytochrome P450 4G1	Likely control anti-bacterial activity through modulation of cytochrome P450 4G1, however, the mechanism requires further investigation	(57)
			dme-miR-133-3p	Down ↓	MAP kinase transcripts and spätzle	Likely negatively regulate the activity of spätzle to regulate immune responses	(57)
Drosophila	*E. coli*	Adult flies	miR-317	Up ↑	PGRP-LC	Following infection, Relish simultaneously increases the production of *Diptericin* and miR-317. The miR-317 upregulation can suppress PGRP-LC expression, thereby inhibiting over-activation and restoring immune homeostasis	(58)
*A. pisum*	*M*. *luteus* and *P*. *aeruginosa*	Aphids whole body	miRNA-184 (miRNA-184a/b)	Down ↓	JNK-3	These results demonstrate that JNK is targeted and negatively regulated by miRNA-184a/b in pea aphid	(59)

↑ upward arrow indicates that the expression of miRNA induced. ↓ Downward arrow shows that the expression of miRNA reduced. ? question mark shows that relevant information is still undiscovered.

### 5.1 Bacillus thuringiensis

*Bacillus thuringiensis*, or Bt, is a microbial, biological insect control agent widely used worldwide because it is an effective means of controlling insects. The Bt bacterium produces crystal proteins by which it poisons, paralyzes, and kills the targeted pests after they have been ingested. A considerable amount of Bt is found in soils across all types of terrain, from desert to tundra, throughout the world. This is a soil-dwelling bacterium that is used to control various pest species. In the context of Bt resistance, over 30 generations of exposure to Bt spores and endotoxins in *G. mellonella*, which resulted in about an 11-fold increase in Bt resistance, resulted in differential expression of miRNAs and, in turn, the suppression of potential genes that confer susceptibility to Bt in the insect (60). Two independent studies have now shown a difference in the expression of miRNAs between Bt-resistant versus Bt-susceptible strains of *O. furnacalis*, with many of these miRNAs predicted to target receptor genes of Bt, including aminopeptidase N and cadherin (61, 62). In a recent study, small RNA libraries were constructed from the midguts of the Cry1Ac resistant (Cry1S1000) strains and the Cry1Ac susceptible (G88) strains. From these libraries, 437 miRNAs were isolated, including 76 known miRNAs and 361 novel miRNAs. These miRNAs were then categorized into 91 families. The expression of 12 miRNAs differed between the Cry1S1000 and G88 strains. In the Cry1S1000 strain, nine miRNAs were suppressed, while three were enhanced when compared to the G88 strain. The miRNAs have been described to regulate cellular processes, metabolism, membranes, and catalytic activity, and the Hippo, MAPK signaling pathway may play a biological role in Bt resistance. Following that, using dual luciferase reporter assays, it was confirmed that novel miR-240, one of the miRNAs with differential expression and a negative correlation with its target genes, interacted with both (53) Px007885 and Px017590 (63).

Furthermore, a recent study challenged *P. xylostella* with *B. thuringiensis* to analyze its impact on *P. xylostella* miRNA expression patterns. According to this study, miRNA profiles were analyzed using RNA sequencing at a range of time points (6, 12, 18, 24, and 36 h), and a combined set of 149 miRNAs was identified following filtration of data. Based on the miRNA profiles, the authors suggested that miRNAs play a biological role in insect immunity in response to bacterial pathogens (53). Interestingly, after being exposed to *B. thuringiensis*, strong expression of a few conserved miRNAs was reported, including miR-1, miR-10, miR-184, miR-275, and Let-7. Following the infection, there was also an increase in the expression of the miR-2, a conserved miRNA. Additionally, miR-2b-3p mimics have the ability to remarkably down-regulate the expression of their target gene, trypsin, which suggests that it may play an important function in the defense mechanism of a lepidopteran species, *P. xylostella*, against an infection caused by *B. thuringiensis* (9, 53).

### 5.2 Micrococcus luteus

*Micrococcus luteus* is a Gram-positive, obligatory aerobic, cocci-shaped bacteria that has been found in water, air, soil, and in the dust. It is a commensal micro-organism of the mouth, upper respiratory tract, and skin; however, in immunocompromised individuals, it may be pathogenic (64, 65). Wei et al. (46) treated *Drosophila* with *M. luteus* and tested miRNA levels at 3, 12, and 24 h time points. They discovered a total of 93 miRNAs, which had differential expression across three-time points. Interestingly, the number of miRNAs that showed differential expression increased in abundance as infection time progressed. This is because it was not until 24 h after infection that the majority of differentially expressed miRNAs were found to exhibit differential expression. Thus, it appears that the number of differentially expressed miRNAs showed an up-ward trend during the induction of the immune response of *Drosophila*. The number of differentially expressed miRNAs at 3 and 12 h time points was remarkably lower than the number of differentially expressed miRNAs at 24 h time points. Moreover, the authors discovered that there were four miRNAs that were considerably enhanced, two miRNAs that were remarkably repressed at 3 h, as well as seven miRNAs that were remarkably increased, and seven miRNAs that were suppressed at 12 h; and 60 miRNAs and 26 miRNAs were downregulated after 24 h. It is also critical to point out that only miR-979-3p had a significant upregulation after 3 and 24 h, and miR-1017-3p had considerable upregulation after 12 and 24 h; while miR-317-3p had a downregulation after both 12 and 24 h; whereas miR-375-3p had a downregulation at 12 h and an upregulation at 24 h. Overall, these findings have shown that the number of miRNAs with differential expression increases during the immune response of *Drosophila* to an infection of *M. luteus*, and that their production trends differ considerably between the early stages (3 and 12 h) and late stage (24 h) of infection with *M. luteus*. This suggests that dynamic expressions of miRNA may play important biological functions in the immune response of Drosophila to an infection caused by *M. luteus*. In response to bacterial infections, the dynamic miRNA-mRNA regulatory network showed that the dynamic miRNAs were associated with the regulation of innate immunity, development, neurogenesis, and memory formation; however, the miRNAs associated with the immune response regulator intensity increased with the infection time (46).

### 5.3 Wolbachia pipientis

*Wolbachia pipientis* has been reported to occur in a broad range of terrestrial arthropods, and it is predicted that it can be found in between 40% and 65% of insects that are currently known to exist (66, 67). This intracellular bacterium, *W. pipientis*, preferentially infects the gonadal tissue cells of insects, disrupting the reproductive cycle of insects (68, 69). Some Wolbachia strains have also been shown to be capable of inhibiting the replication of various RNA viruses. This phenomenon is being used to reduce mosquito-borne pathogenic virus transmission (67, 70, 71). It seems that transinfected Wolbachia, which are not naturally present in the *Aedes aegypti* mosquito depend on host and endosymbiont adjustments for the Wolbachia to persist. Several lines of evidence suggest that Wolbachia dysregulates the regulation of miRNAs in its insect hosts (54, 72). Microarray analysis of wMelPop-transinfected female *Ae. Aegypti* has shown that 13 mosquito miRNAs were altered in abundance as a result of trans-infection. The results of experimental studies in which a number of these differentially abundant miRNAs were manipulated resulted in the conclusion that they play a critical role in maintaining the growth and survival of Wolbachia. When the expression of aae-miR-2940, which has been found to be highly expressed in mosquitoes that are infected with Wolbachia, is suppressed using an inhibitor of aae-miR-2940, the density of Wolbachia in mosquitoes also decreases (54). *In vitro* analysis has shown that manipulation of three miRNA targets, methyltransferase 2 (73), metalloprotease FtsH (54), and arginine methyltransferase (74), resulted in a decrease in Wolbachia density. As a result, this miRNA and its target genes are likely to play a crucial biological role in the replication and persistence of Wolbachia in mosquito cells. It was also observed that the inhibition of the aae-miR-12 expression, which is activated by wMelPop-infected female *Ae. aegypti*, also reduced the density of Wolbachia in cells that were persistently infected with the endosymbiont. A recent study investigated the differentially expressed miRNAs in Wolbachia-infected *Laodelphax striatellus*. It was found that Wolbachia infection upregulated 18 miRNAs and suppressed six miRNAs in males, whereas 25 miRNAs were enhanced and 15 miRNAs decreased in females. Additionally, it was demonstrated that the target genes of these miRNAs that had differential expression were involved in the regulation of immune response, redox homeostasis, ecdysteroidogenesis, and reproduction (72). It was interesting to note that the miRNA expression pattern of a *D. melanogaster* cell line (JW18) that had been treated by the wMel strain, Wolbachia, did not change as a result of the treatment. Additionally, RT-qPCR analysis of an Aag2 cell line that had been infected by the wMel strain revealed that there were no variations in the abundance of a number of miRNAs that were tested (75). This lack of change in miRNA levels might be explained by the fact that miRNAs were analyzed in cell lines *vs.* whole organisms and by the fact that there were variations between strains of Wolbachia (wMel *vs.* wMelPop, is a strain that is extremely pathogenic and attains higher densities). Furthermore, the latter observation is also supported by the fact that aae-miR-2940 was likewise upregulated in *Ae. albopictus* C6/36 cells that were exposed to wMelPop in comparison to uninfected C6/36 cells (54). Asad et al. also reported dramatic variations in the tissue level aae-miR-2940 abundance in the wMelPop mosquitoes and tetracycline-treated mosquitoes (55).

Furthermore, it has been suggested that Wolbachia wMelPop has the ability to change the distribution of Ago1 between the nucleus and cytoplasm, which in turn has the effect of influencing the localization of miRNAs in both the nucleus and the cytoplasm. This is accomplished by changing the abundance of miRNAs in *Ae. aegypti* and the distribution of miRNAs in the nucleus (76). In addition, miRNA isomers can be altered, which may result in changes to their target gene repertoire (77). There was also evidence of miRNA-like small RNAs released into the cytoplasm of the host cell by the endosymbiont from Wolbachia wMelPop, which could have an effect on target genes within the host cells. In addition to being positively regulated by one of these Wolbachia miRNA-like small RNAs,WsnRNA-46, in mosquitoes infected with Wolbachia, Dynein heavy chain (Dhc) is also expressed at higher levels in those infected with Wolbachia. A previous study demonstrated that microtubules, along with Dynactin and Dynein proteins that are associated with them, are used by Wolbachia for cellular localization and movement in *D. melanogaster* oocytes, are pivotal to the normal levels of Wolbachia and can be involved in the effective transmission of Wolbachia through the mother (78).

### 5.4 Buchnera aphidicola

*Buchnera aphidicola*, a gamma proteobacterium that has an obligatory relationship with aphids and plays a fundamental biological role in their nutritional metabolism and development (79–81). In a recent study, it was found that five miRNAs, including miR-10, miR-184a, miR-276, miR-3050, and bantam, had high expression levels in the bacteriomes of the aphid *Myzus persicae* (29). The results of a study that compared the miRNA expression profiles from bacteriomes and guts, which do not contain Buchnera bacteria, revealed that 17 miRNAs have different levels of abundance in these two types of tissues; this also includes the five miRNAs that have a high level of transcription in both types of tissues. In addition to the three miRNAs that were only found in *M. persicae*, the remaining 14 miRNAs were detected to have differential expression in *M. persicae* lineages, as well as *Aphis pisum*, which is one of the other aphid species. The findings indicate that these miRNAs most likely play an important role in the interaction between the endosymbiont and their aphid hosts, as well as their biological roles may be conserved across aphid species. The researchers linked the orthologs of ten of the 14 miRNAs to interactions between insect hosts and pathogens (29). Because Buchnera provides the host with amino acids that are both essential and non-essential, it was suggested that genes that contributed to amino acid metabolism and transport would be overrepresented among the possible targets of these 14 miRNAs (80). In addition, target gene profiling revealed that a number of signal transduction pathways are involved in the interaction between Buchnera and aphids, which suggests that these pathways may facilitate symbiosis.

### 5.5 *Listeria* monocytogenes

*Listeria monocytogenes*, an intracellular bacterium, is a common human pathogen, and its outbreak cause severe economic losses in terms of organismal deaths (82, 83). There is also evidence that this bacterium infects insects and influences their miRNAs (57, 84). A recent study systematically analyzed changes in the expression levels of miRNA in *G. mellonella* larvae after they were infected with *L. monocytogenes*. This study found that infection with *L. monocytogenes* influenced the expression patterns of a total of 90 miRNAs, 39 of which were enhanced and 51 miRNAs repressed. Non-pathogenic *L. innocua*, on the other hand, failed to trigger the transcription of these miRNAs, suggesting a virulence-dependent miRNA dysregulation. The differentially expressed miRNAs that were discussed earlier were shown to contribute to autophagy, innate immunity, and signal transduction, such as optineurin, MAP kinase, and spätzle, respectively, all of which displayed a virulence-specific differential expression (57).

### 5.6 Pseudomonas entomophila

It is a unique feature of *P. entomophila* among the other species of Pseudomonas because of its ability to naturally infect and kill insects upon ingestion. Since its discovery in 2005, *P. entomophila* has emerged as one of the highly imperative models for investigating interactions that exist between insects and microbes in recent years. It was primarily obtained from a single female *D. melanogaster* that had been collected in Calvaire (Guadeloupe) as a part of screening that was designed to identify bacteria that cause infection in *Drosophila*, and at the time, it was named strain L48T. Following ingestion of this strain, a systemic immune responses were induced in *D. melanogaster* of both larval and adult stages (85, 86). The red flour beetle *T. castaneum* fed with the bacterial entomopathogen *P. entomophila*, injected with peptidoglycan or treated to either starvation or mild heat shock. *P. entomophila* was shown to have affected 455 mature arthropod miRNAs. Feeding *T. castaneum* with *P. entomophila* and injecting the beetles with peptidoglycan were likewise found to have altered the abundance of the miRNAs in the beetles. The treatment with *P. entomophila* strongly induced at least seven miRNAs in the insect; however, oral uptake of this bacteria led to the suppression of 11 miRNAs, whereas only three were enhanced (87).

### 5.7 Serratia entomophila

*S. entomophila* is a bacterial species that belongs to the genus Serratia and has been identified as an entomopathogenic bacteria. This species can be isolated using the selective caprylate thallous agar. A study carried out on *G. mellonella* larvae that were fed on diets contaminated with either *S. entomophila* or *E. coli* has demonstrated the transcription of particular miRNAs in the egg’s midgut and rest of the body. The latter caused api-miR-263a to be specifically upregulated, whereas *S. entomophila* resulted in miRNA being specifically downregulated. Api-miR-263a governs a large number of down-stream targets, and the oppositional reactions to various organisms indicate that certain transcriptomic processes are organized against pathogens like *S. entomophila* in comparison to *E. coli*. There has been a report that larval diets that were contaminated with *S. entomophila* can induce particular immune responses in both the guts of larvae and in the eggs laid by insect females that consumed these bacteria as larvae during their life time, suggesting specific immune priming across generations (56, 88). It has also now been established that the production of api-miR-263 occurs in the midguts of larvae that have been fed with *S. entomophila* bacteria as well as in the eggs of females who have been fed with these pathogenic bacteria when they were in the larval stage of their life. It appears that api-miR-263 has a biological role in immune priming across generations (60).

### 5.8 Other bacteria responsible for miRNAs regulators in insects

In addition to the above-mentioned bacterial pathogens, miRNA expression patterns have also been influenced by many other bacterial pathogens in insects. For example, Lourenco et al. (89) collected honey bee *Apis mellifera* samples after infecting them with *Serratia marcescens* and assessed the expression levels of various miRNAs in *A. mellifera*: miR-12, miR-2, miR-34, miR-184, miR-13a, miR-92a, miR-375, miR-278, miR-1175, miR-1006, and miR-989, let-7, bantam. Previously, Chen et al. (90) demonstrated a low transcription level of miR-1006, which suggest that this miRNA exhibits spatial or temporal expression trends. Additionally, most of the miRNAs studied by Lourenco et al. (89) were down-regulated in bees injected with bacteria (ame-miR-1175, ame-miR-184, ame-miR-375, ame-miR-34, ame-miR-12, ame-miR-989, ame-miR-278, and ame-bantam), whereas some were induced (ame-miR-13a, ame-miR-92a, ame-let-7, and ame-miR-2). However, only miR-1175 and miR-13a exhibit considerable variation in mRNA levels after infection that was caused by *S. marcescens*. The dysregulation profiles of particular miRNAs during the duration of the infection suggest that these miRNAs are the critical regulator of the immune system. Uropathogenic *Escherichia coli* (UPEC) strains are the ones that are responsible for causing symptomatic urinary tract infections in humans. On the other hand, commensal, like *E. coli* strains that live in the urinary bladder, is the ones that are responsible for causing asymptomatic bacteriuria on a long-term basis. *G. mellonella*, is a surrogate host insect model that is used to investigate human pathogens like UPEC (91). The sequencing of miRNA in larvae of *G. mellonella* that had been infected with either ABU strain 83972 or UPEC strain CFT073 displayed substantial variations in the production levels of miRNAs in *G. mellonella* larvae. Based on these results, it appears that immune response-mediated miRNAs of insects are able to differentiate between pathogenic and commensal *E. coli* invasions (92).

## 6 Autophagy

The autophagy process is an extremely conserved biological mechanism in which damaged organelles and self-proteins are hydrolyzed via autophagosomes in order to degrade. In addition to this, it plays a variety of host-pathogen interactions and is a component of the cellular immune response of host to a pathogen. There is a well-established fact that pathogens are capable of manipulating autophagy responses to both improve the rate at which they replicate as well as establish infection within the host (3, 11). The modulation of miRNAs, along with changes in the autophagy-associated factors, indicates that miRNAs seem to control the process of autophagy (11, 57). Optineurin is one of the most conserved receptors for autophagy in eukaryotes, and it plays a critical biological role in the removal of bacteria from the intracellular environment (93, 94). According to the results of a study conducted on the insect *G. mellonella* (a powerful infection model), optineurin was strongly induced following bacterial exposure, and this was associated with a reduction in the regulatory miRNAs expression, including miR-998 and miR-133 (57). The results of this study suggest that this interaction may facilitate in the increased clearance of intracellularly localized pathogens by autophagy. Additionally, Mannala et al. (57) reported that the activation of these signaling pathways seems to be virulence dependent. This is due to the fact that miR-133 and miR-998 and their target transcripts did not appear to be deregulated through infection with *L. innocua*, except for the MAP kinase protein, which was induced to a lesser degree upon infection. The removal of *L. innocua* may be associated with the expression of other proteins, such as lysozyme2 and chitotriosidase-1, both of which have been shown to be upregulated in *L. innocua* infections but downregulated in *L. monocytogens* infections (95). It is likely that pathogenic bacteria suppress chitotriosidase-1 and lysozyme2 as a mechanism of evading the host-response, whereas nonpathogenic pathogens, including *L. innocua*, are effectively eliminated by the activity of lysozyme2. Indeed, the bmo-miR-3000 (may actively be involved in autophagy) has been shown to be upregulated during infection with *L. monocytogenes* and downregulated during infection with *L. innocua*, as it was shown to (57). So far, only a few studies have discussed the biological role of miRNAs in the removal of bacterial pathogens ***via*** autophagy in insects. More research on this topic is needed to understand the precise mechanism by which miRNAs play a role in autophagy processes.

## 7 Immune signaling is affected by miRNA fluctuation during bacterial infection

**Figure 3:**
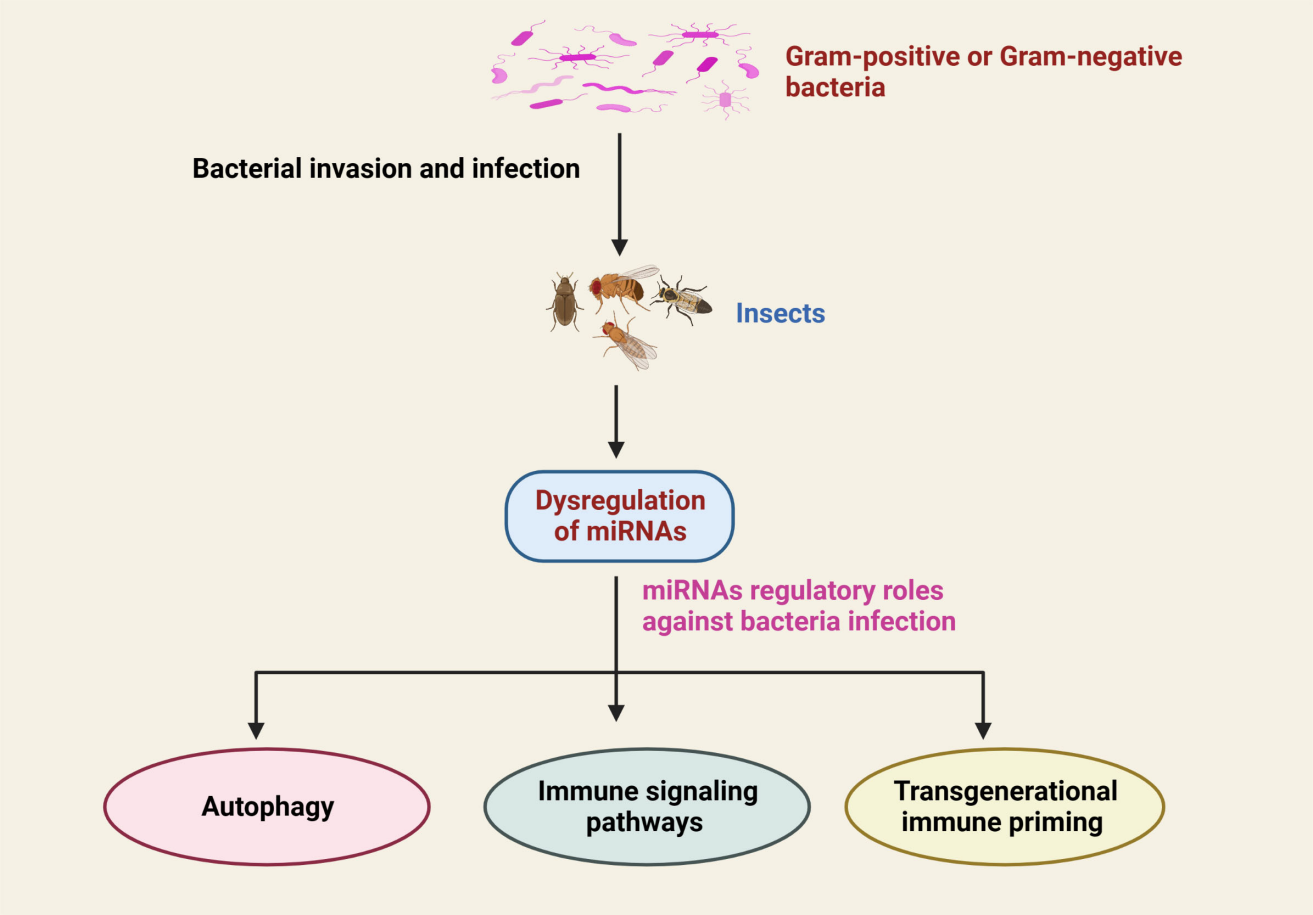
Schematic representation of the dysregulation of insect miRNAs in response to infection with Gram-positive or Gram-negative bacterial infection and their critical regulatory functions in the prevention of bacterial infection.

It has been established that in order for the host to respond effectively to microbial pathogens, a number of cellular signals require to be finely modulated, including immune signaling (3, 96–98). As described above, miRNA expression is strongly altered in response to bacterial pathogens. Thus, it has been proposed that altered miRNA may influence various pathological processes and physiological functions in insects. The modification of physiological activities, however, is dependent on the type of miRNAs and their target genes (**Table 1**; **Figure 3**).

### 7.1 MiRNAs regulate the Toll signaling pathway in insect

**Figure 4:**
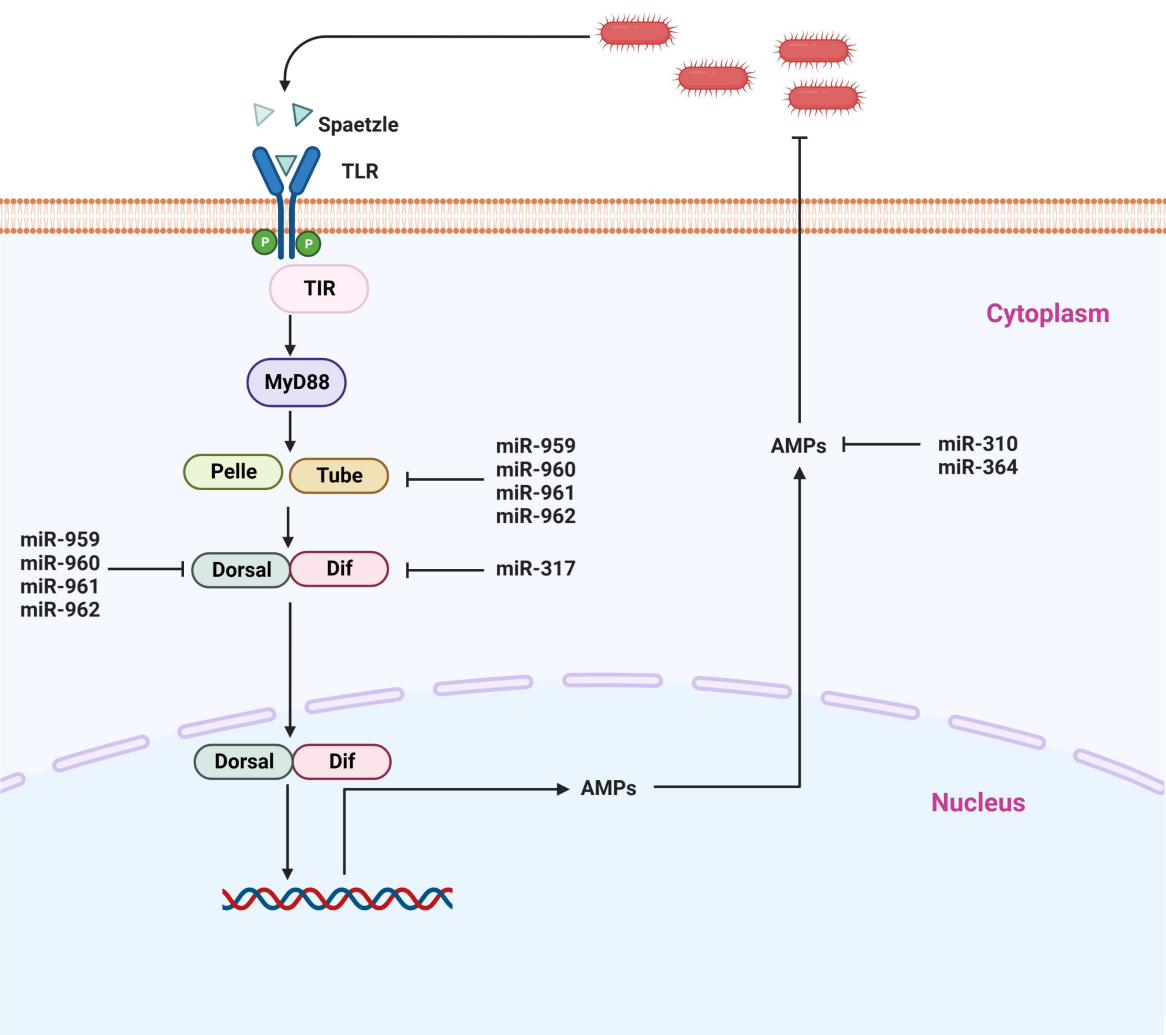
Representative microRNAs that control the Toll signaling pathway. Toll receptors recognize various bacterial components and either activate NF-κB signaling or induce other transcription factors *via* adapter molecules and downstream signaling molecules.

The Toll signaling pathway is considered one of the major pathways involved in the counter-infection of Gram-positive bacteria and other microbial pathogens such as fungi and viruses (99–101). The activation of this signaling pathway occurs when Lys-type peptidoglycan as well as the β-1, 3-glycan of Gram-negative bacteria enter the cell, resulting in the proteolytic cleavage of the proSpätzle molecule (102, 103). On the plasma membrane of the cell, Spätzle binds to the Toll receptor (104), and as a result, this complex induces a signal transduction cascade in the cytoplasm by its interaction with the myeloid differentiation primary response protein (MyD88)-Tube-Pelle complex. It is through this process that Pelle phosphorylates and degrades Cactus, resulting in the release of Dorsal and Dif (105). In recent studies, there has been growing evidence that when the NF-kB family members Dif and Dorsal are translocated into the cell nucleus, it triggers the production of various antimicrobial peptides such as drosomycin, defensin 2, metchnikowin, and others (**Figure 4**) (106).

In insects, it has recently been shown that besides coding sequences, miRNAs have also played a crucial biological role in the modulation of immune homeostasis by regulating the Toll signaling pathway (47, 107, 108). So far, most of the studies addressing the association of miRNAs have been performed on model insects, particularly *Drosophila*. It has been demonstrated that there are two mechanisms by which miRNAs are capable of governing the activity of the Toll signaling pathway: either individually or in a synergistic manner. It is believed that miRNAs suppress the levels of key components of immune signaling in the host when they are infected. Li et al. (9) identified four miRNA members from the miR-959-962 cluster (miR-959/miR 960/miR 961/miR 962) as novel suppressors of Toll signaling, and they also found that each of the four miRNA members contributed to reducing antimicrobial peptide drosomycin expression and the survival rate of *Drosophila*. According to the findings of their investigation, dorsal and tube mRNAs that essential components of the Toll signaling pathway can simultaneously be targeted by miR-959, miR-960, miR-961, and miR-962, respectively. Even more specifically, miR-962 is capable of directly targeting the 3’ UTR of the Toll pathway. In addition, these four miRNA members have been found to be an important player in the immune homeostasis restoration of *Drosophila* at the late stages of *M. luteus* infection, suggesting that miR-959-962 cluster plays a crucial role in the immune response of *Drosophila* to *M. luteus* infection *via* negatively regulating the Toll signaling pathway resulting in a decreased survival rate of *Drosophila via* suppressing the production of AMPs (9). It was further shown in the same study that miR-960 is capable of modulating anti-bacterial defense only after the 12 h stage after infection of bacteria. It may be possible that miR-959, at the same time, is constantly suppressing the expression of Dorsal at two different time points (6 and 12 h). On the other hand, miR-961 may contribute more to repressing antibacterial defense than miR-962. Also, in a previous study, miR-958 was isolated and identified in-silico strategy using the Gal80ts-Gal4 driver system, and it was found to be associated with innate immune responses of the fly. It has been shown that miR-958 is an important miRNA candidate that is capable of potentially modulating the Toll signaling pathway both *in vivo* and *in vitro*, by negatively targeting both Toll and Dif, which inhibits the Drosomycin expression in *Drosophila*. There is a strong indication that miR-958 inhibits Toll transcription specifically and significantly at its site 3, as the Toll 3’ UTR possesses four miR-958-binding sites (109). In a similar manner, *Drosophila* miR-317 suppressed only the Dif-Rc, which is one of four isoforms that participate in the Toll signaling pathway, in an attempt to negatively regulate *Drosophila* Toll signaling response (48). A previous study by the same authors has demonstrated that miR-317 controls the Toll signaling pathway in *Drosophila* by targeting the three additional Dif isoforms (Dif-Ra, Dif-Rb, and Dif-Rd) (48, 109). It has been noted that flies that overexpress miR-317 transiently have poor survival. On the other hand, the knockout miR-317 flies (miR317 KO/+) show better survival during Gram-positive bacterial infection in comparison to the control group (48), suggesting that miRNA plays a role in the crosstalk between immunity and survival in *Drosophila*. Furthermore, it has been reported that four members of the *Drosophila* miR-310 family, including miR-310, miR311, miR-313, and miR-312, are negatively modulating the Toll-mediated immune functions in *Drosophila.* They accomplish this by inhibiting the production of Drosomycin and directly co-targeting the 3’UTR of Drosomycin in *Drosophil*a that has been infected with Gram-positive bacteria (110). Another study has shown that miR-964 deficiency in *Drosophil*a results in hyperactivation of AMP gene *Drosomycin*, which increases the survival rate of flies when challenged with *M. luteus*, while miR-964 over-expression compromises innate immunity in flies. Only the 3′-UTR of *Drosophila Drosomycin* is the direct target gene of miR-964, as other Toll pathways associate antimicrobial peptides (Defensin and Metchnikowin) showed no change. Thus, it seems that miR-964 does not inhibit the production of other components in Toll signaling, which suggests that miR-964 fine-tunes the regulatory circuit that is required to maintain the homeostasis of the Toll pathway by suppressing the expression of the AMP gene directly (50). The intron of CG31646 is responsible for the encoding of the miR-964, which is a member of the miR-959-964 cluster. The miR-959–964 cluster was shown in a previous study to either the peak survival time or inhibit immune function against pathogen infection (111). In spite of the fact that the molecular mechanism that underlies the miR-959–964 cluster appears to be rather complicated, it is abundantly clear that miR-964 becomes one of the regulators in the Toll pathway. In addition to this, the miR-959–964 cluster demonstrated a consistent phase and a strong amplitude. Therefore, it is likely that they are encoded in just a single transcription unit. It is important to take note of the fact that members of the miR-959–964 cluster each have their own unique seed sequence. Furthermore, it was found that the miR-959–964 cluster has a more remarkable impact on the Toll signaling pathway than does miR-964 alone. As a result, determining the potential immune role of each member of the cluster in relation to the pathogen would be an interesting endeavor. However, the number of immune genes that are targeted by the miR-959–964 cluster is still unknown, as is the degree to which each miRNA participates in the maintenance of immune homeostasis of *Drosophila.* In order to resolve this ambiguity, additional research needs to be conducted (**Figure 4**) (50).

On the whole, besides the coding sequences, insects also comprise miRNAs, which appear to play their functional role either individually or collectively. Based on the above studies, it is evident that miRNAs are the main player in the negative regulation of the Toll pathway and consequently repress the production of antimicrobial peptides, which are important for the attenuation of bacterial infection. Thus, miRNAs are involved in controlling overshooting of immune responses, thereby regulating immune homeostasis. The vast majority of this information is derived from the model insect Drosophila; therefore, further research is required to identify miRNAs and determine the molecular mechanism of these miRNAs in relation to the Toll pathway. Hence, in order to further improve our understanding of miRNA’s molecular functions, we should cover a wide range of insect species in our research.

### 7.2 MiRNAs regulate IMD signaling pathway in insects

**Figure 5:**
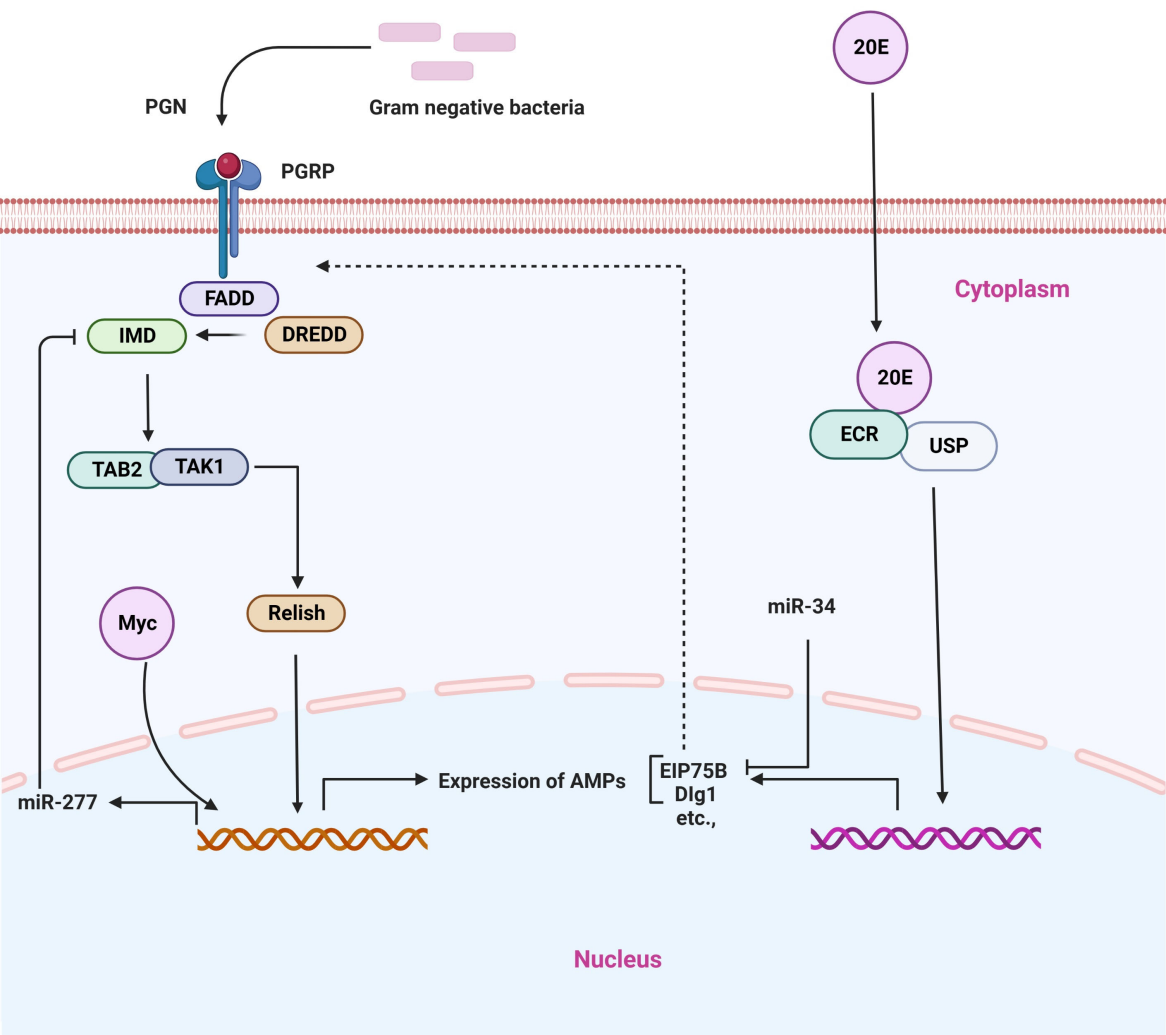
Representative microRNAs in the regulation of IMD signaling pathway. PGRP receptor recognizes different bacterial components and activates the downstream signaling cascade.

Immune deficiency (IMD) is a conserved signaling pathway in insects and other animals that has the ability to activate NF-κB. This pathway plays a key biological function in immune homeostasis *via* regulating the expression of AMPs during pathogen infection in insects. Thus, this signaling pathway seems indispensable for the regulation of immune responses (2, 3). In general, when Gram-positive bacteria invade the host, they activate the PGRP-LC receptor that is located on the membrane of cells. This leads to the activation of the Imd pathway, which in turn promotes the cleavage of Relish into Rel-N (Rel68). Rel-N (Rel68) can then translocate into the cell nucleus to induce the transcription of AMPs, including Diptericin and Attacin-A (**Figure 5**) (112).

There is mounting evidence that miRNAs are important regulators of the IMD signaling pathway (51, 113). Like in the case of other biological processes, miRNAs play an important role in the IMD pathway by repressing the expression of target genes in ways that are sequence-specific manner. In a model insect (*Drosophila*), the conserved miRNA, miR-34, regulates innate immunity. It has been shown that miR-34 over-expression can activate anti-bacterial immune signaling both *in vivo* and in cultured cells and flies that over-express miR-34 exhibit increased survival and pathogen clearance following infection with Gram-negative bacterial pathogens. On the other hand, miR-34 knockout insects are found to be defective in their ability to defend against bacterial infection. In particular, it has been shown that miR-34 can regulate the IMD pathway in part through repressing genes that encode specific proteins that are known to be associated with septate junctions, such as Dlg1, and Eip75B, which are components of the ecdysone signaling cascade and a negative modulator of the IMD signaling pathway (51). Recently, another study has isolated and identified the *Drosophila* Myc gene (a broad-spectrum transcription factor), which is a negative modulator of the IMD pathway in *Drosophila*. The *Drosophila* Myc transcription factor is positively activating miR-277 expression, thereby inhibiting the expression of IMD and Tab2-Ra/b by targeting their 3’UTR, which helps to maintain immune homeostasis. Importantly, *Drosophila* Myc is able to improve the survival rate of flies after they have been infected, which suggests that repressing the IMD pathway of *Drosophila* through *Drosophila* Myc is critical to restoring immune homeostasis, which is required for the survival of flies. Thus, it appears that the *Drosophila* Myc-miR-277-imd/Tab2 axis is engaged in the negative modulation of the IMD pathway in *Drosophila*; thereby, this axis regulates the mechanism of *Drosophila* innate immune homeostasis maintenance (52). Furthermore, another miRNA, miR-317, is involved in the regulation of the IMD pathway. Relish can directly trigger miR-317 production to target PGRP-LC in addition to AMPs, so generating a negative feedback loop that facilitates to restoration of immune homeostasis during the activation of the IMD pathway in *Drosophila*. Mechanically, in *Drosophila*, during the infection of Gram-positive bacteria (*E. coli*) stimulate various gene expression, in particular, the Dpt, in which expression levels is increased at 3 h and reach a peak at 12 h. During the process of a short immune response, both the Relish and the PGRP-LC receptor, which actively responds to the invasion of *E. coli* are also greatly produced in the early stage (3 h) of innate immunity, exhibiting the rapid immune response of the innate immunity. Interestingly, the dynamic expression of miR-317 also shows a similar trend to that of Dpt, suggesting that Relish improves the production levels in a cell. There is evidence to suggest that miR-317 produces relatively high levels of transcript roughly during early infection time (6–12 h), which indicates this miRNA has some other unknown function in fighting against Gram-negative bacterial infection. Thus, it appears that Relish plays an important function in the process of activating the immune system, but it also plays a crucial role in the process of restoring immune homeostasis, which is dependent on the function of miR-317. Collectively, miR-317 forms an axis with other effectors that is known as the Relish/miR-317/PGRP-LC axis to restore immune homeostasis and negative feedback regulation of the IMD immune response in *Drosophila*. Additionally, miR-317 has the ability to suppress the over-activation of IMD immune functions and to restore immune homeostasis (**Figure 5**) (58).

In summary, it is important to note that miRNAs have received less attention as regulators of the IMD signaling pathway in insects. However, few studies have explored the miRNA functional role as regulators of the miRNA pathway, which has previously been discussed. Furthermore, the exact molecular mechanisms for these miRNAs have not been determined, necessitating further research in order to understand the exact functions and molecular mechanisms of these miRNAs, as well as to identify the tissues that contain these miRNAs, as well as the sites where the IMD pathway is activated to fight infections.

### 7.3 MiRNAs govern the JNK signaling pathway in insects

Insects, being the most diverse group of animals, occupy almost all different types of environmental conditions (2, 13). However, most insect species are equipped with defense systems that protect them in unfavorable conditions. These insect species contain a conserved set of genes that encode immune effectors. The immune responses of hemipteran species, including the *Acyrthosiphon pisum* aphid, are considerably suppressed in comparison to those of holometabolous insect species, which display a lower level of immune responses. The genome-wide analysis of the hemipteran showed that the pea aphid does not contain genes that encode for scavenging receptors, PGRPs, AMPs, IMD, and other immune-associated molecules (59, 114). Besides most of the immune effector molecules, only the Jun N-terminal kinase pathway (JNK) was reported to be present in the pea aphid, and that is mainly involved in the immune responses against various pathogens (114). It has recently been shown by Ma et al. (59) that this signaling pathway plays a pivotal biological role in modulating the immune system of pea aphids when they are invaded by bacterial pathogens. The study further found that miRNA-184 targeted the JNK-3’UTR and inhibited its transcription, thereby providing a favorable environment for a bacterial proliferation in the aphids and resulting in an increase in the mortality of the aphids after infection. There is a significant drop in miRNA-184b and miRNA-184a expression following the infection caused by *Pseudomonas aeruginosa* and *M. luteus*, with the lowest level of expression being detected within 24 h after the infection (59). This may be due to a negative correlation between JNK expression and miRNA-184a and miRNA-184b expression. It is very interesting to note that prophenoloxidase activity, phagocytosis, and reactive oxygen species in the pea aphid are all controlled by the JNK signaling pathway, indicating that miRNA-184 governs these anti-bacterial immune responses in an indirect manner. There is also evidence to suggest that miRNA-184 regulates the JNK signaling pathway in a universal manner, as prediction using the RNA hybrid program demonstrated that JNK is likely a target of miRNA-184 in insect species, other invertebrates, and vertebrates (52).

## 8 MiRNA involve in transgenerational immune priming

In vertebrates, females achieve transgenerational immune priming (TGIP) by passing antibodies to their offspring. It is important to note that certain insects, like vertebrates, have a process known as immune priming, which is an effective survival strategy for these species. A sub-lethal dose of a microbial pathogen, or material derived from a pathogen, has been reported to induce an immune response in insects, rendering them more resistant to a subsequent lethal infection within a short period of time after immune priming. It is generally believed that this biological process is mediated through an increase in the density of circulating hemocytes and an increase in the level of AMPs (115). It has recently been shown that miRNAs may be involved in the process of TGIP in insects. According to Freitak et al. (87), the miRNAs play an important biological role in regulating immunity-regulating genes, both sex-specific and stressor-dependently, in *T. castaneum* beetle. The convergent transcriptional reprogramming of immune-related genes and genes involved in stress-response, along with the large array of miRNAs, has led to the discovery of an important role for miRNAs. *G. mellonella* api-miR-263 has been identified as the first miRNA to be linked to TGIP in insects. It has been shown that upregulation of this miRNA is associated with the guts of larvae that have been exposed to entomopathogenic bacteria, *S. entomophila*, as well as the eggs laid by females that have been exposed to the bacteria as larvae (116). There is, therefore, a possibility that in insects, miRNAs could facilitate paternal TGIP by being delivered in the sperm (117).The interesting thing is that despite the fact that there is still a great deal of work to be done regarding the miRNAs involved in TGIP, this is the only plausible molecular mechanism that is available for paternal TGIP. The environmental-induced epigenetic (e.g., miRNAs) transgenerational inheritance of sperm epigenetic marks may also cause genetic mutations in the offspring as a result of pathogen-triggered epigenetic marks (118). This suggests that pathogen-induced epigenetic changes, particularly miRNA marks, might also be mediating TGIP in insects in order to benefit the offspring of these insects. It is also important to note that it is still unclear whether miRNAs, which considered to promote transgenerational immune priming are actually transported from individuals who have been exposed to a pathogen to the subsequent generation.

## 9 Conclusion and perspectives

In the past few years, research on the miRNA has provided an unprecedented opportunity for us to investigate the mechanisms by which the innate immune system detects pathogens and responds to them in response to the infection they cause. The biological role of miRNAs in infection that is caused by a bacterial pathogen has led to significant advances in our understanding of cellular physiology and immunology over the last decade. Our review article aims to provide a detailed overview of the wide range of effects that different miRNAs may have on the immune system and the molecular mechanisms that govern the production of these miRNAs in the tissues of insects. Based on the findings, which provide new highlights on how the miRNA pathway evolved in insects and other animals; secondly, we discussed the various types of bacteria that cause infection and how those bacteria influence the expression profiles of miRNAs in diverse insect species; thirdly, we highlighted the molecular mechanism that host miRNAs use to regulate cellular immunity in response to bacterial pathogens; following that we described how host miRNAs govern different signaling pathways that have been established to be effective against bacterial infections; and finally we focused on the biological role that host miRNAs play in the process of immune priming. Moreover, a large number of other miRNAs have been identified as differentially expressed during infection that are predicted to target immunity-related genes (49, 109, 110, 119) or have been demonstrated to influence immune activity but have yet to be discovered (49, 51, 52, 110, 120, 121). There is a need to investigate the precise mechanism by which miRNAs can regulate the activity of cells. Additionally, there is a need for further research in order to gain a better understanding of the effects that miRNAs with a range of targets have on the overall responses of the host during an infection. It has been observed that, in some cases, the continuous progression of an infection process is accompanied by alterations in the transcription level of miRNA as a consequence of the continuous progression of the infection process. There is a need to differentiate which regulators are the most imperative in this regard. The multiple virulence factors developed by bacterial pathogens are thought to be responsible for their ability to invade and multiply within their hosts, invade tissues, and evade host defense systems. So far, researchers have only discovered bacterial infections that modify the transcription levels of a large number of miRNAs. Many different types of miRNAs could be induced during bacterial infection. More research is required to determine the specificity of miRNA transcription triggered through specific bacterial pathogens. In order to understand the complex relationship between miRNAs and pathogenic bacteria, as well as the mechanisms that underlie this relationship, more research is urgently required. Therefore, it may be possible to develop novel and more effective prevention strategies based on a deeper understanding of the biological role miRNAs play during host-pathogen interactions.

## Author contributions

The authors’ responsibilities were as follows: MA and SK designed this review article. MA, SK, and BA downloaded the material and wrote down the draft. WR, JL, and ZL drew the diagram. TL and HC proofread the article. All authors contributed to the article and approved the submitted version.
